# Prognostic value of multi-PLD ASL-based cerebral perfusion ASPECTS in acute ischemic stroke

**DOI:** 10.3389/fneur.2024.1476937

**Published:** 2024-10-09

**Authors:** Qingqing Li, Chaojun Jiang, Linqing Qian, Jing Yang, Tianchi Mu, Congsong Dong, Shu Wang, Zhenyu Wang, Hengheng Liu, Yijun Dong, Zhenyu Dai, Fei Chen

**Affiliations:** ^1^Department of Radiology, Suzhou Wuzhong People's Hospital, Suzhou, Jiangsu, China; ^2^Department of Radiology, Affiliated Hospital 6 of Nantong University, Yancheng, Jiangsu, China; ^3^Department of Radiology, The People's Hospital of Danyang, Affiliated Danyang Hospital of Nantong University, Danyang, Jiangsu, China; ^4^Department of Ultrasound, Affiliated Hospital 6 of Nantong University, Yancheng, Jiangsu, China; ^5^Department of Radiology, Affiliated Yancheng Third People's Hospital of Jiangsu Vocational College of Medicine, Yancheng, Jiangsu, China

**Keywords:** acute ischemic stroke, Alberta Stroke Program Early CT Score, arterial spin labeling, cerebral blood flow, cerebral blood volume

## Abstract

**Introduction:**

We aimed to verify the application value of the Alberta Stroke Program Early CT Score (ASPECTS) based on multiple post-labeling delay (multi-PLD) arterial spin labeling (ASL) for outcome assessment in acute ischemic stroke (AIS) patients.

**Method:**

The endpoint was modified Rankin scale score at 90 days (90-day mRS). Patients were divided into the good outcome (0–2) and poor outcome (3–6) groups. The independent samples *t*-test, Mann-Whitney *U*-test, and χ^2^-test were used to compare clinical and imaging parameters between groups. We used partial correlation analysis to evaluate the relationships between ASPECTS and outcomes. Multivariate logistic regression analysis was used to examine potential independent prognostic indicators. The receiver operating characteristic (ROC) curve analysis was used to evaluate the performance of the independent prognostic indicators in predicting outcomes.

**Results:**

Fifty-five AIS patients were included. The good outcome group had a lower baseline National Institutes of Health Stroke Scale (NIHSS; *Z* = −3.413, *P* < 0.001) and infarct core volume (ICV; *Z* = −3.114, *P* = 0.002) as well as higher cerebral blood flow (CBF)-ASPECTS (*Z* = −3.835, *P* < 0.001) and cerebral blood volume (CBV)-ASPECTS (*Z* = −4.099, *P* < 0.001). Higher CBF-ASPECTS (*r* = −0.459, *P* = 0.001), and CBV-ASPECTS (*r* = −0.502, *P* < 0.001) were associated with a lower 90-day mRS. The baseline NIHSS, CBF-ASPECTS, and CBV-ASPECTS were identified as independent prognostic indicators. The AUCs of the baseline NIHSS, CBF-ASPECTS, and CBV-ASPECTS were 83.3, 87.4, and 89.9%, respectively. Combining NIHSS with CBF-ASPECTS and CBV-ASPECTS, the AUC significantly improved to 96.3%. The combined three factors showed a significant difference compared to the baseline NIHSS (*Z* = 2.039, *P* = 0.041) and CBF-ASPECTS (*Z* = 2.099, *P* = 0.036), but no difference with CBV-ASPECTS (*Z* = 1.176, *P* = 0.239).

**Conclusions:**

The ASPECTS based on multi-PLD ASL is a valuable tool for identifying independent prognostic indicators and assessing clinical outcomes in AIS patients. The baseline NIHSS, combined with CBF-ASPECTS and CBV-ASPECTS, enhances the predictive efficacy of clinical outcomes in AIS patients. The CBV-ASPECTS alone can offer comparable predictive efficacy to the combination.

## 1 Introduction

Acute ischemic stroke (AIS) is now the most fatal condition in China, and its global impact is on the rise (1, 2). Permanent damage to brain cells can occur within minutes of losing blood supply. The most successful way to rescue patients and reduce their disabilities is to rapidly restore the flow of blood to the brain (3, 4). A growing attention has been paid to cerebral perfusion, particularly represented in quantitative parameters, as a strong association has been found between the presence of good brain perfusion and favorable radiological and clinical outcomes in patients with AIS (5).

Arterial spin labeling (ASL) is a non-invasive imaging approach that enables quantitative measurements of cerebral perfusion in AIS patients (6). However, the accuracy can be affected by several factors, the most significant being the applied post-labeling delay (PLD) (7). For different populations, the duration for hydrogen protons in the blood to progress from the initiation of the labeling procedure to the location of collection, i.e., the arterial transit time (ATT) may be dissimilar. Earlier studies utilizing single-PLD ASL have been observed to either overestimate or underestimate cerebral blood flow (CBF) (8, 9). Multiple acquisitions can reduce bias, and obtain additional information, such as cerebral blood volume (CBV) and ATT (10). However, it also increases time costs. Recently, multiple post-labeling delay (multi-PLD) ASL has been shown to improve signal-to-noise ratio (SNR) and temporal efficiency as a single-acquisition, time-coded quantitative technology (11–14). In several trials, multi-PLD ASL has been proven to be accurate and consistent in measuring cerebral perfusion in healthy adults and children (15–17). To summarize, this imaging method requires a shorter scanning duration but improves the accuracy, sensitivity, and reliability of cerebral perfusion (18–21).

The Alberta Stroke Program Early CT Score (ASPECTS) provides a simple means of evaluating early ischemic changes in the middle cerebral artery (MCA) blood supply area of individuals with AIS (22). ASPECTS was originally designed for unenhanced CT images. However, the ideal ASPECTS would be to assess the area of early ischemic changes on a perfusion map to identify the core of the infarct (23). Computed tomography perfusion and magnetic resonance perfusion imaging are considered the benchmark for assessing cerebral perfusion. However, their applications were restricted due to its invasive nature and the potential risk of allergic reactions to contrast agents. Thus, examining the ASPECTS based on ASL is highly significant. Nevertheless, there is a scarcity of research on the correlation between ASPECTS determined by ASL and the prognosis of ischemic stroke. Currently, there are only two studies that rely on single-PLD ASL and solely employ visual artificial analysis on the CBF parameter map (24, 25). However, both the assessment of CBF by single-PLD ASL and the CBF-ASPECTS based on visual were lacking in accuracy. Therefore, we hypothesized that the ASPECTS based on multi-PLD ASL could detect imaging independent prognostic indicators of AIS patients, and was an effective tool for prognostic assessment.

In this study, we used multi-PLD ASL to quantitatively assess CBF, ATT, and CBV in the brain tissue of AIS patients with non-major vessel occlusion. The ASPECTS of CBF, ATT, and CBV were calculated. Combined with clinical data, we analyzed whether perfusion ASPECTS were correlated with the clinical outcome of AIS patients, whether they were independent prognostic indicators, and their efficacy in predicting the clinical outcome.

## 2 Materials and methods

### 2.1 Patients

**Figure 1:**
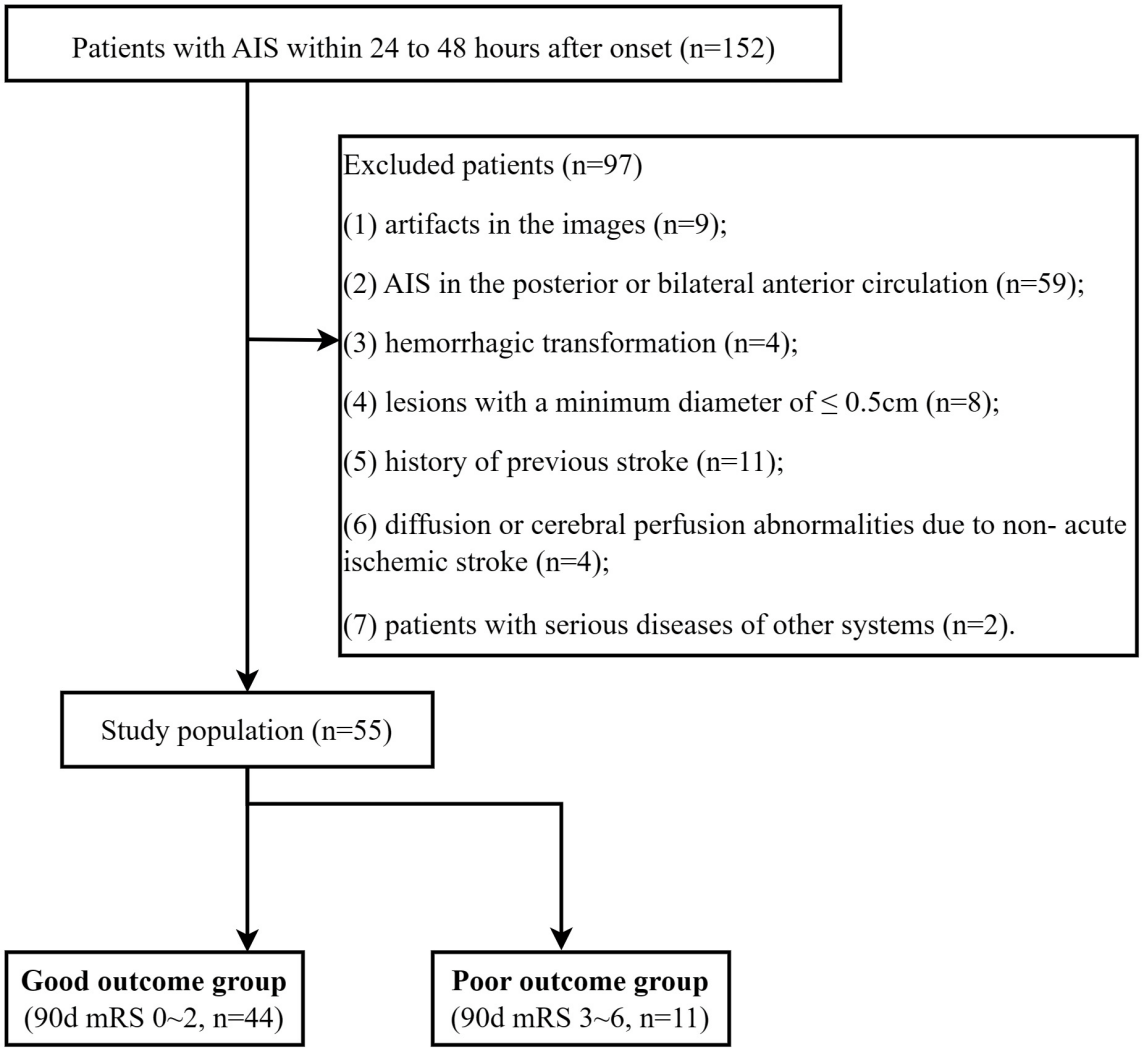
Study flow diagram.

A total of 55 AIS patients (36 men [65.45%]; mean [SD] age, 66.61 [11.56] years) were enrolled between December 2020 and March 2022 (Figure 1). All patients underwent MRI examinations within 24–48 h after symptom onset. The trial was approved by the ethics committee of Yancheng Third People's Hospital (Ethics No. 2020-77). All participants in this trial provided informed consent.

The inclusion criteria were as follows: (1) a confirmed diagnosis of AIS according to the guidelines (26); (2) patients who have exceeded the time window and cannot be treated with thrombolysis or embolization; (3) adults aged 18 or older.

The exclusion criteria were as follows: (1) images with artifacts; (2) AIS in the posterior or bilateral anterior circulation; (3) hemorrhagic transformation; (4) lesions with a minimum diameter of ≤ 0.5 cm; (5) a history of previous stroke; (6) diffusion or cerebral perfusion abnormalities due to non-acute ischemic stroke; (7) patients with serious diseases of other systems.

### 2.2 Clinical assessment

All participants received standard medical therapy, including antiplatelet aggregation, anticoagulation and lipid lowering. Every patient received a neurological score based on the National Institutes of Health Stroke Scale (NIHSS; range, 0–42, with higher scores indicating greater deficit) on admission. The clinical and demographic parameters of the patient were noted from the electronic medical record (Table 1), including age, sex, history of atrial fibrillation, hyperlipidemia, diabetes mellitus, hypertension, alcohol abuse, and smoking. According to the modified Rankin Scale score at 90 days (90-day mRS), scores range from 0 to 6, with scores higher than 2 indicating a poor outcome. Therefore, patients were divided into the good outcome group (0–2) and the poor outcome group (3–6).

**Table 1:** Demographic, clinical characteristics, and ASL parameters of the good and poor outcome groups.

**Characteristic**	**Good (*n* = 44)**	**Poor (*n* = 11)**	***t*/*Z*/χ^2^**	***P*-value**
Age, year^a^	68 ± 11	75 ± 11	−1.986	0.052
Male sex, *n* (%)	28 (63.64)	8 (72.73)	0.322	0.571
ICV, ml^b^	6.71 (2.09, 19.89)	11.67 (6.96, 31.76)	−3.114	0.002^*^
**Risk factors**, ***n*** **(%)**				
Atrial fibrillation	6 (13.64)	2 (18.18)	0.146	0.702
Hypertension	36 (81.82)	11 (100)	2.340	0.126
Diabetes mellitus	15 (34.09)	1 (9.09)	2.666	0.102
Hyperlipidemia	20 (45.45)	6 (54.55)	0.292	0.589
Smoking history	12 (27.27)	4 (36.36)	0.353	0.553
Alcohol abuse	11 (25)	2 (18.18)	0.227	0.634
NIHSS^b^	3 (1, 6)	8 (4, 13)	−3.413	<0.001^*^
**ASL parameters**				
CBF-ASPECTS^b^	7 (5, 9)	3 (2, 4)	−3.835	<0.001^*^
CBV-ASPECTS^b^	7 (6, 9)	4 (2, 4)	−4.099	<0.001^*^
ATT-ASPECTS^b^	10 (10, 10)	10 (9, 10)	−1.772	0.181

n, number of cases.

^a^($\bar{\chi }\pm$s).

^b^[M (Q1, Q3)].

NIHSS, baseline National Institutes of Health Stroke Scale; ASPECTS, Alberta Stroke Program Early CT Score; ICV, infarct volume; CBF-ASPECTS, CBF-based ASPECTS; CBV-ASPECTS, CBV-based ASPECTS; ATT-ASPECTS, ATT-based ASPECTS.

The marked with “^*^” is statistically significant (P <0.05).

### 2.3 MR imaging protocol

In this trial, MRI examination was conducted using a 3 T unit (Discovery 750, GE Healthcare, Waukesha, WI, USA) with a 24-channel head and neck coil. The routine MRI protocol included T_2_-weighted imaging (T_2_WI; TR: 4,357 ms, TE: 1,000 ms), T_2_-based fluid attenuation inversion recovery (T_2_WI-FLAIR; TR: 9,000 ms, TE: 140 ms), T_1_-weighted imaging (T_1_WI; TR: 2,213.1 ms, TE: 77.2 ms), diffusion weighted imaging (DWI; TR: 3,586 ms, TE: 77.2 ms, b: 1,000 s/mm^2^), 3D time-of-flight (TOF) MRA (TR: 20 ms; TE: 3.4–5 ms). Multi-PLD ASL was simulated with the following parameters: PLD_1 − 7_ = 1.0, 1.22, 1.48, 1.78, 2.1, 2.63, 3.32 s; TR: 5,978 ms, TE: 11.5 ms, FOV: 22 cm × 22 cm, thickness: 4.5 mm, number of layers: 106, resolution: 4.67 mm × 4.67 mm, NEX: 1, scan duration: 4 min 2 s.

### 2.4 Imaging assessment

The infarct core was defined as the area with ADC <620 (× 10^−6^ mm^2^/s), and the infarct core volume (ICV) was automatically obtained by Astroke software (version 1.0, Animage Beijing Technology Co, Ltd) based on the ADC map (27, 28).

**Figure 2:**
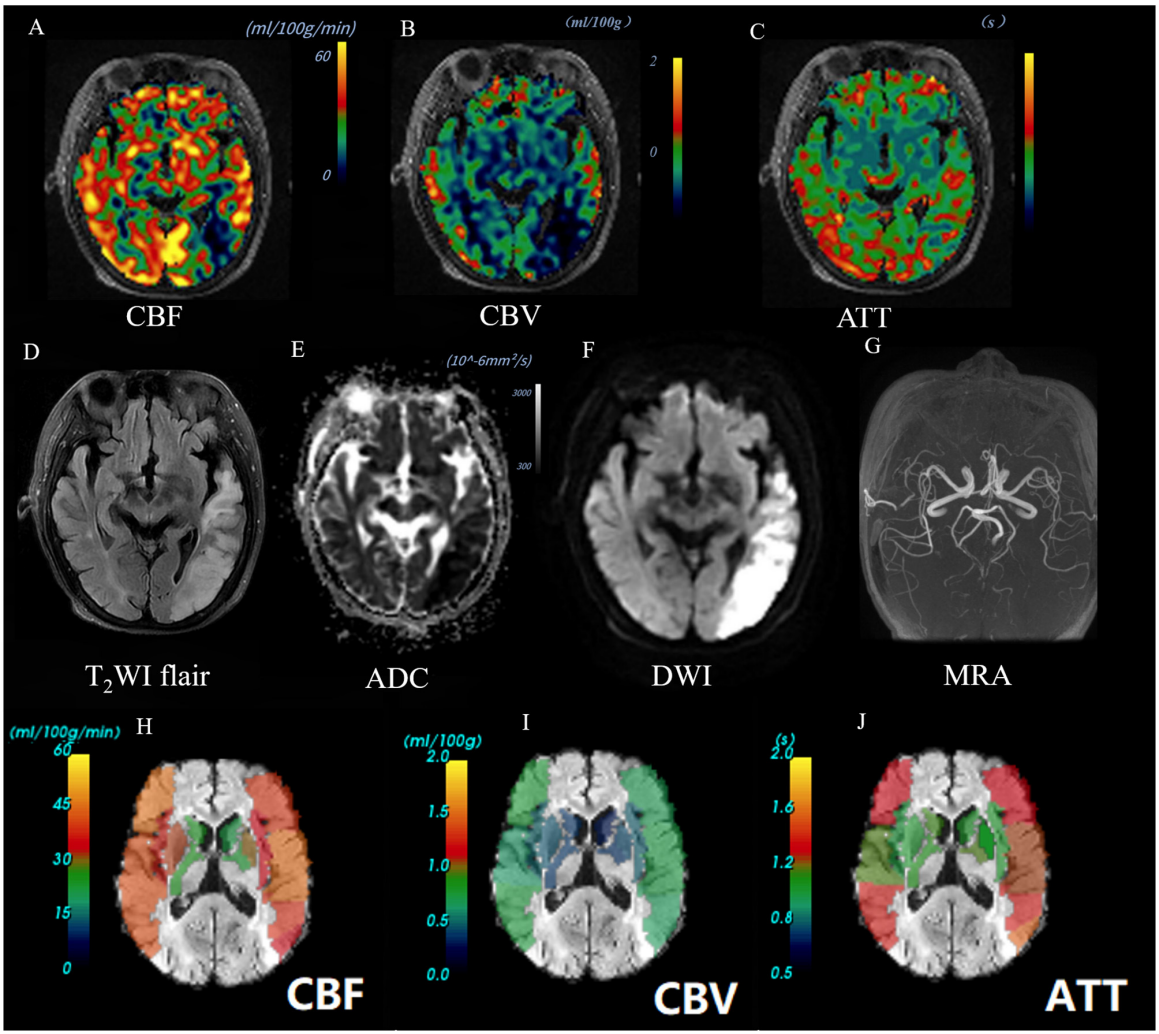
Images of an AIS patient with a good outcome (90-day mRS = 0). A 66-year-old female patient was diagnosed with AIS in the left temporal lobe. Baseline NIHSS score of 1 point, 90-day mRS score of 0 point. The CBF perfusion map **(A)** and CBV perfusion map **(B)** showed low perfusion of the lesion. The ATT perfusion map **(C)** revealed an insignificant change. The lesion showed high signal on T_2_Flair **(D)** and DWI map (b = 1,000) **(F)**, and the ADC value decreased **(E)**. The 3D-TOF-MRA map showed no obvious stenosis or occlusion **(G)**. The ASPECTS template was used to standardize the perfusion parameters across 10 different zones. **(H–J)** The scores obtained were as follows: CBF-ASPECTS: 8 points, CBV ASPECTS: 9 points, ATT-ASPECTS: 10 points. Warm-to-cold color bars represent values from high to low.

**Figure 3:**
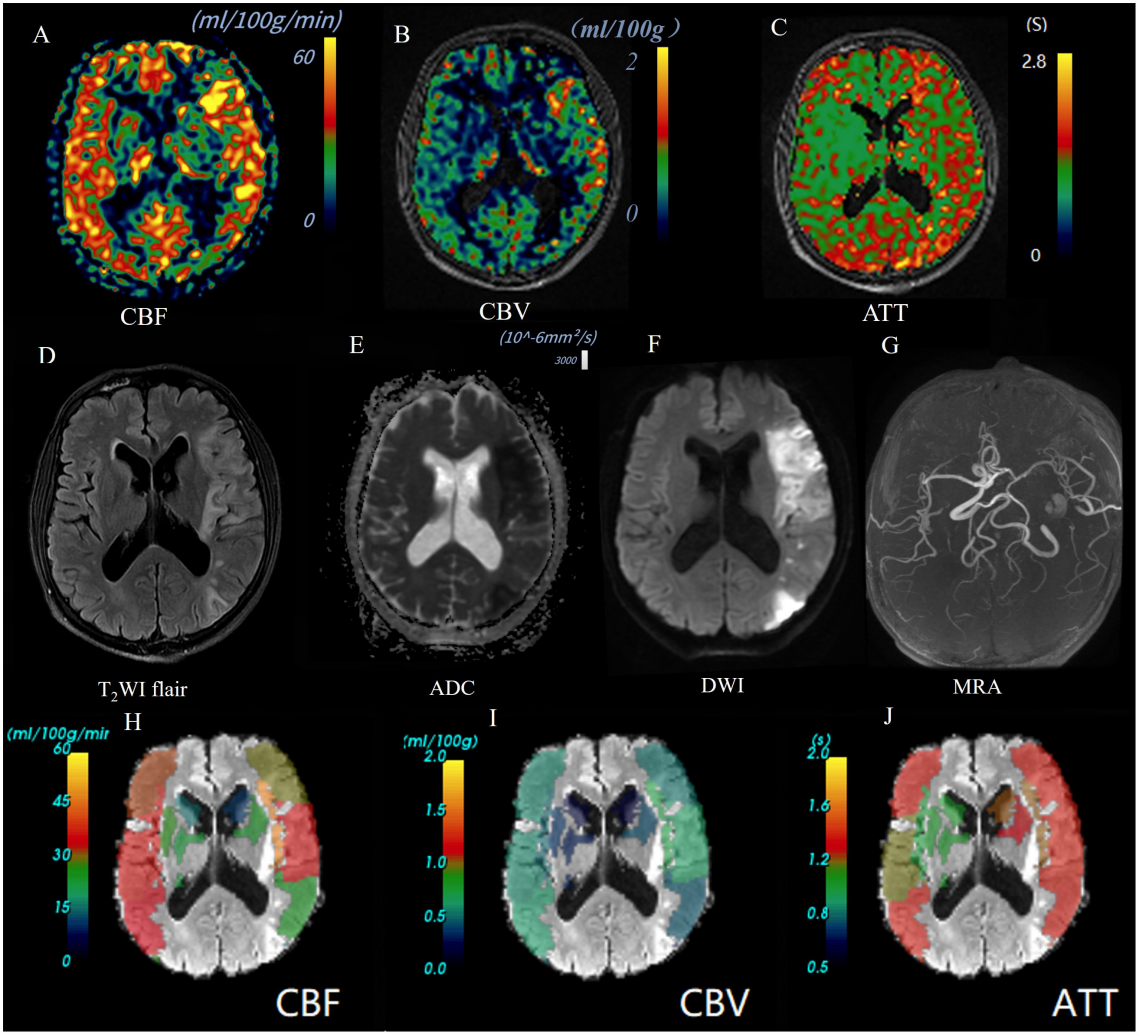
Images of an AIS patient with a poor outcome (90-day mRS = 4). A 55-year-old male patient was diagnosed as AIS in left temporal parietal occipital lobe. Baseline NIHSS score 10 points, 90 d mRS score 4 points. CBF perfusion map **(A)** and CBV perfusion map **(B)** showed high perfusion of the lesion; ATT perfusion map revealed extended arrival time **(C)**. The lesion showed high signal on T2Flair **(D)** and DWI map (b = 1,000) **(F)**, and the ADC value decreased **(E)**. 3D-TOF-MRA map showed the left middle cerebral artery is narrow but not occluded **(G)**. Warm-to-cold color bars represent values from high to low. The ASPECTS template was used to standardize the perfusion parameters across 10 different zones. **(H–J)** The scores obtained were as follows: CBF-ASPECTS: 6 points, CBV ASPECTS: 9 point, ATT-ASPECTS: 8 points.

Multi-PLD ASL data were quantitatively analyzed through an automatic software CereFlow (http://www.cereflow.cn; Animage Beijing Technology Co, Ltd; Figures 2, 3). The processing steps were as follows: Firstly, physiological noise and motion correction were performed between the marker and control pictures, followed by pairwise subtraction (29). Using a conventional non-linear iterative curve fitting method based on the traditional single-chamber perfusion model, mean perfusion maps of multi-PLD ASL were generated. These maps were based on quantitative values of CBF and CBV, which were corrected for ATT. The SimpleITK (http://www.simpleitk.org) normalization tool was applied to the estimated perfusion images to generate the ATT, CBF, and CBV values for ASPECTS. The difference index of perfusion between the left and right brain regions was calculated, using the quantification algorithm given as follows:


(1) $$\begin{array}{l} \frac{\left(L-R\right)}{\left(L+R\right)}2. \end{array}$$

The regions where the difference index ≥0.2 were considered to be regions with ischemic changes (24). Based on the above formula and thresholds, we recorded the number of regions with cerebral ischemic changes in the ASPECTS brain regions and calculated ASPECTS based on CBF, CBV, and ATT, respectively, by subtracting the number of regions from 10.

All participants in this study underwent imaging evaluations by two neuroradiologists (F.C. and C.J.J.), each with at least 10 years of experience in MR imaging. Conflicts were resolved through consensus, and the mean level was measured for statistical analysis.

### 2.5 Statistical methods

Analyses were performed using SPSS software (Version 26, IBM, Armonk, NY, USA) and MedCalc software (Version 20, Belgium, GER). To compare the variables of the good outcomes group and the poor outcomes group, we employed independent samples *t*-test or Mann-Whitney *U*-test (non-normal distribution) for continuous variables and χ^2^-test for categorical variables. The relationship between ASPECTS and clinical outcomes (90-day mRS) was analyzed using partial correlation analysis. Based on the between-group comparison results, binary logistic regression was utilized to investigate potential independent prognostic indicators, with a *p*-value selected at *P* < 0.1. Due to the collinearity among the ASPECTS of perfusion parameters, it is necessary to develop logistic regression models separately with clinical data for conducting multi-factor analysis. The receiver operating characteristic (ROC) curve was used to evaluate the effectiveness of individual prognostic indicators or their combination in differentiating AIS patients with different clinical outcomes. The DeLong test was used to statistically compare the areas under the curves (AUC). Statistical significance was defined as *P* < 0.05.

## 3 Results

### 3.1 Clinical and imaging comparison between the good outcome group and the poor outcome group

The images of AIS with different outcome were shown in Figures 2, 3. Compared to the poor outcome group, the good outcome group had a lower baseline NIHSS (*Z* = −3.413, *P* < 0.001), ICV (*Z* = −3.114, *P* = 0.002), and higher CBF-ASPECTS (*Z* = −3.835, *P* < 0.001), and CBV-ASPECTS (*Z* = −4.099, *P* < 0.001; Table 1). There were no significant differences in age, sex, atrial fibrillation, hypertension, diabetes, hyperlipidemia, smoking, alcohol abuse, or ATT-ASPECTS between the two groups (all *P* > 0.05).

### 3.2 Correlation between ASPECTS of perfusion parameters and clinical outcome

**Figure 4:**
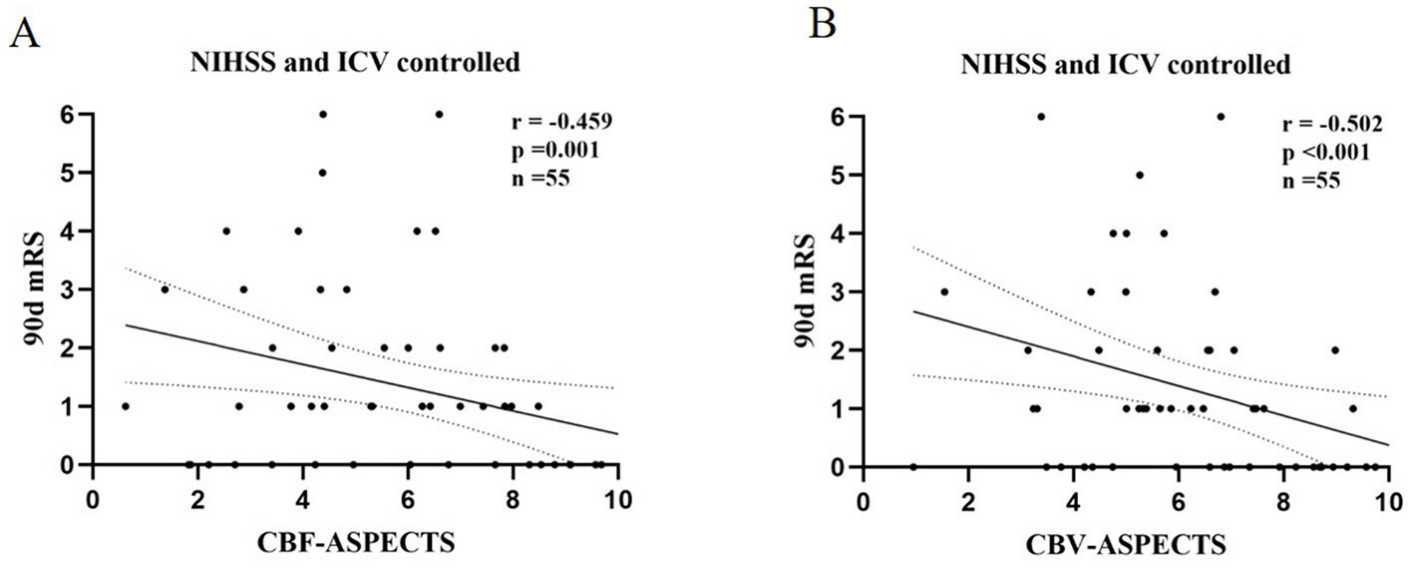
The partial correlation analysis of CBF-ASPECTS **(A)** / CBV-ASPECTS **(B)** and clinical outcome (90-day mRS) with baseline NIHSS and ICV controlled.

With the partial correlation analysis, the clinical outcome (90-day mRS) showed separate negative correlations with CBF-ASPECTS (*r* = −0.459, *P* = 0.001), and CBV-ASPECTS (*r* = −0.502, *P* < 0.001), correcting for baseline NIHSS and ICV as covariates (Figure 4). Nevertheless, there was no correlation found between ATT-ASPECTS and clinical outcomes (*r* = −0.099, *P* = 0.482).

### 3.3 Multivariate logistic regression analysis of prognostic indicators

Potential prognostic indicators including age, ICV, baseline NIHSS, CBF-ASPECTS, and CBV-ASPECTS were identified based on the comparison between groups with different clinical outcomes. Due to the collinear relationship between CBF-ASPECTS and CBV-ASPECTS, two logistic regression models were constructed separately. Model 1 (Table 2) showed that the baseline NIHSS (OR = 1.67, *P* = 0.023) and CBF-ASPECTS (OR = 0.32, *P* = 0.016) were independent prognostic indicators. Model 2 (Table 3) showed that the baseline NIHSS (OR = 1.55, *P* = 0.030) and CBV-ASPECTS (OR = 0.41, *P* = 0.016) were independent prognostic indicators.

**Table 2:** Multivariate logistic regression analysis of independent prognostic indicators (model 1).

**Prognostic indicators**	**B**	**S.E**.	**Wald χ^2^**	**Exp (B)**	**95% CI**	***P*-value**
Age	0.048	0.058	0.697	1.05	0.94–1.18	0.404
NIHSS	0.511	0.226	5.136	1.67	1.07–2.60	0.023^*^
ICV (ml)	0.047	0.036	1.720	1.05	0.98–1.13	0.190
CBF-ASPECTS	−1.131	0.469	5.812	0.32	0.13–0.81	0.016^*^

NIHSS, baseline National Institutes of Health Stroke Scale; ASPECTS, Alberta Stroke Program Early CT Score; ICV, infarct core volume; CBF-ASPECTS, CBF-based ASPECTS.

The marked with “^*^” is statistically significant (P <0.05).

**Table 3:** Multivariate logistic regression analysis of independent prognostic indicators (model 2).

**Prognostic indicators**	**B**	**S.E**.	**Wald χ^2^**	**Exp(B)**	**95% CI**	***P*-value**
Age	0.056	0.064	0.761	1.06	0.93–1.20	0.383
NIHSS	0.436	0.202	4.653	1.55	1.04–2.30	0.030^*^
ICV (ml)	0.028	0.029	0.922	1.03	0.97–1.10	0.337
CBV-ASPECTS	−0.895	0.372	5.780	0.41	0.20–0.85	0.016^*^

NIHSS, baseline National Institutes of Health Stroke Scale; ASPECTS, Alberta Stroke Program Early CT Score; ICV, infarct core volume; CBV-ASPECTS, CBV-based ASPECTS.

The marked with “^*^” is statistically significant (P <0.05).

### 3.4 The predictive efficacy analysis of independent prognostic indicators

**Figure 5:**
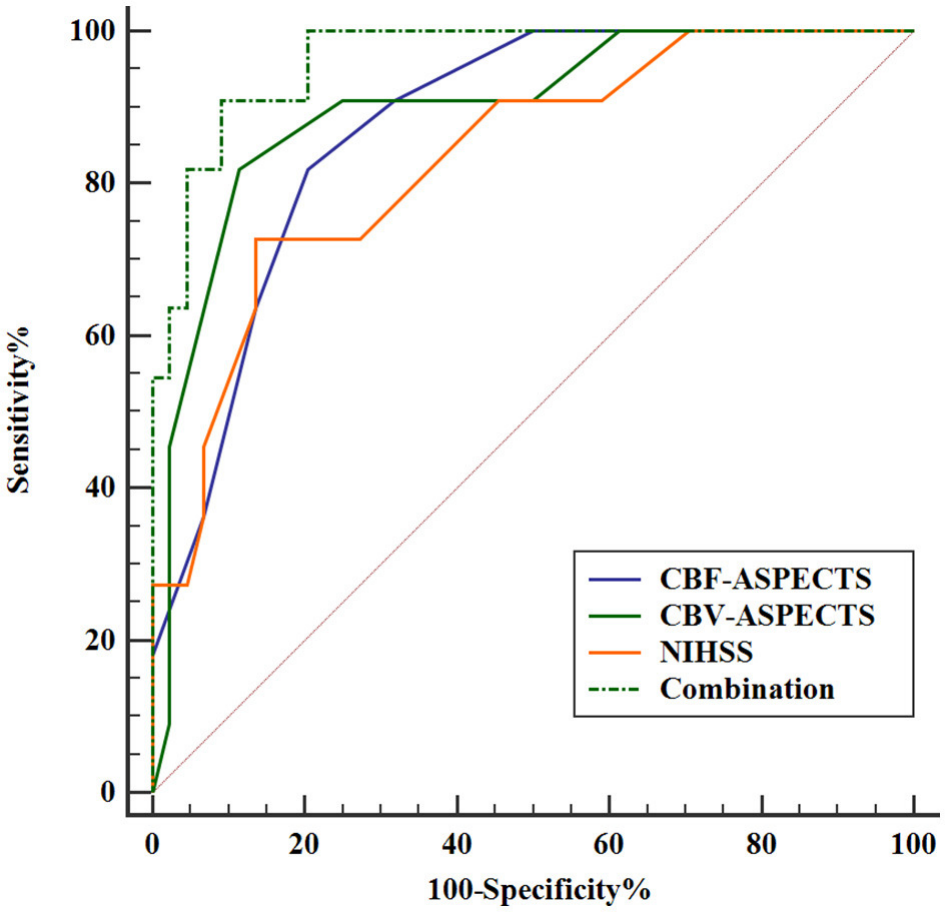
ROC curves analysis of independent prognostic indicators and their combination for predicting clinical outcomes.

The Receiver Operating Characteristic (ROC) analysis was employed to evaluate the potential of independent prognostic indicators and their combination in accurately distinguishing the different clinical outcomes of AIS patients (Figure 5). Table 4 showed that the baseline NIHSS had an AUC of 83.3% [*P* < 0.001, 95% confidence interval [CI] = 0.698–0.968, with a cut-off value of 3]; the CBF-ASPECTS had an AUC of 87.4% (*P* < 0.001, 95% CI = 0.777–0.971, with a cut-off value of 8); and the CBV-ASPECTS had an AUC of 89.9% (*P* < 0.001, 95% CI = 0.793–1.000, with a cut-off value of 6). The sensitivity of the baseline NIHSS, CBF-ASPECTS, and CBV-ASPECTS were 0.727, 0.818, and 0.818, and the specificity were 0.864, 0.795, and 0.886, respectively. When adding CBF-ASPECTS and CBV-ASPECTS to the baseline NIHSS, the AUC significantly improved to 96.3% (*P* < 0.001, 95% CI = 0.916–1.000). Both sensitivity and specificity were increased to 0.909. The combination of the three indicators was found to have a significant difference compared to the baseline NIHSS (*Z* = 2.039, *P* = 0.041), CBF-ASPECTS (*Z* = 2.099, *P* = 0.036), but no difference with CBV-ASPECTS (*Z* = 1.176, *P* = 0.239).

**Table 4:** ROC curves analysis of independent prognostic indicators and their combination for predicting clinical outcomes.

**Variables**	**AUC**	**SE**	***P*-value**	**95%CI**	**Sensitivity**	**Specificity**	**Youden J**
NIHSS	0.833	0.0493	<0.001	0.698–0.968	0.727	0.864	0.591
CBF-ASPECTS	0.874	0.0540	<0.001	0.777–0.971	0.818	0.795	0.613
CBV-ASPECTS	0.899	0.068	<0.001	0.793–1.000	0.818	0.886	0.704
Combination	0.963	0.023	<0.001	0.916–1.000	0.909	0.909	0.818

NIHSS, baseline National Institutes of Health Stroke Scale; ASPECTS, Alberta Stroke Program Early CT Score; CBF-ASPECTS, CBF-based ASPECTS; CBV-ASPECTS, CBV-based ASPECTS; Combination, combination of NIHSS, CBF-ASPECTS, and CBV-ASPECTS.

## 4 Discussion

This study showed that the baseline NIHSS, ICV, CBF-ASPECTS, and CBV-ASPECTS significantly differed between AIS patients with good or poor outcomes. A higher CBF-ASPECTS or CBV-ASPECTS was associated with a good outcome. Furthermore, baseline NIHSS, CBF-ASPECTS and CBV-ASPECTS were independent prognostic indicators. When adding CBF-ASPECTS and CBV-ASPECTS to the baseline NIHSS, the predictive efficacy of clinical outcome significantly improved. Moreover, the CBV-ASPECTS by itself could offer comparable predictive accuracy to the combination.

The multi-PLD ASL technique is currently one of the most advanced non-invasive imaging methods for detecting brain perfusion (7). By setting multiple PLDs, it avoids the influence of blood flow velocity differences on brain perfusion measurement (13). Multi-PLD ASL has been used in the evaluation of various neurological disorders such as stroke, epilepsy, cognitive impairment, and more. Specifically, diffusion-prepared multi-PLD ASL can be utilized for assessing neurological disorders by detecting the blood-brain barrier function (30, 31). This study leveraged the accurate measurement characteristics of multi-PLD ASL to analyze perfusion changes at the voxel level in patients with AIS. Subsequently, the ASPECTS template was used for localization to analyze the perfusion ASPECTS of these AIS patients.

Consistent with previous trials (32, 33), we found that the good outcome group had a lower baseline NIHSS, ICV. This implies that a smaller baseline injury leads to a better prognosis, which is a logical outcome. A recent study employing multi-PLD ASL found that a higher rCBF, as indicated by ROI analysis, was associated with better early neurological outcomes in AIS patients (6). Manual tracking of ROIs, despite being the most popular method, has many shortcomings. Firstly, ROI-based values are inaccurate, especially when the ROIs contain partial volume effects. Secondly, the infarct areas of stroke patients often contain cortical and gray matter, making it difficult to accurately measure. Thirdly, in longitudinal studies, it is challenging to precisely replicate the same ROI manually, which can also cause bias in the results. In our investigation, we refrained from using the commonly utilized ROI-based analysis method. Instead of that, we employed an analysis method that combines voxel analysis with the ASPECTS template.

In this study, we found that the poor outcome group had lower CBF-ASPECTS and CBV-ASPECTS, and that both CBF-ASPECTS and CBV-ASPECTS were negatively correlated with clinical outcomes (90-day mRS). Lower CBF-ASPECTS based on pseudo-continuous ASL (single-PLD = 2,000 ms) was associated with a poor prognosis for AIS patients, which has been verified in a previous study (24). Moreover, there was a collinear relationship between CBF and CBV, so it is reasonable that there was a consistent rule between CBV-ASPECTS and the prognosis of AIS. To date, there is a lack of studies on the relationship between CBF-ASPECTS and CBV-ASPECTS based on multi-PLD ASL as a more accurate non-invasive perfusion detection method, and the clinical outcome of AIS. In our study, we also found conclusions that are consistent with the study of single PLD ASL. The association between CBF-ASPECTS and CBV-ASPECTS and clinical outcome was observed, with baseline NIHSS and ICV serving as control variables. Our results indicate that there was no statistical difference in ATT-ASPECTS between two groups of AIS patients with different outcomes, and it was not correlated with the clinical outcome. However, ATT has a significant impact on perfusion measurements (12). Additionally, in this study, CBF and CBV measured by multi-PLD ASL were also corrected based on ATT. One possible reason could be that all cases in this group were AIS patients without large vessel occlusion, resulting in no significant changes in ATT and thus no statistical difference between the two prognosis groups. Further investigation is needed to determine the value of ATT-ASPECTS in clinical prognosis assessment of AIS patients with large vessel occlusion. Given the significant correlation between CBF-ASPECTS/CBV-ASPECTS and patient outcomes, multi-PLD ASL could be considered as a non-invasive alternative to CT perfusion for prognostic evaluation, especially in AIS patients with non-major vessel occlusion.

Additionally, the ischemic penumbra is also an important factor influencing the clinical prognosis of AIS patients. Studies have reported that the oxygen extraction fraction map, based on quantitative susceptibility mapping (QSM), holds significant value in detecting the ischemic penumbra in AIS patients (34). Future research can incorporate it into multi-factor prognostic models as a prognostic indicator for analysis. Moreover, studies have shown that the longitudinal changes in magnetic susceptibility values within ischemic lesions based on QSM are significantly related to neurological functional prognosis (35). This provides new insights into exploring non-invasive methods for prognosis assessment in AIS patients.

In our study, independent prognostic indicators were screened from potential prognostic indicators by constructing binary logistic regression models. Given the collinearity between CBF-ASPECTS and CBV-ASPECTS, we constructed two logistic regression models, results shown that CBF-ASPECTS, CBV-ASPECTS, and baseline NIHSS were independent prognostic indicators. The baseline NIHSS serves as an independent prognostic indicator in AIS patients, as evidenced by previous studies (24, 30). Our study was the first to find that CBF-ASPECTS and CBV-ASPECTS based on multi-PLD ASL were independent prognostic indicators in AIS patients. The OR values for CBF-ASPECTS and CBV-ASPECTS were 0.32 and 0.41, respectively, suggesting that the increase in CBF-ASPECTS and CBV-ASPECTS was an inhibiting factor for poor prognosis. It provides a basis for clinical identification of AIS patients with poor prognosis and timely targeted treatment to improve prognosis.

Further analysis of independent prognostic indicators revealed strong predictive efficacy. Baseline NIHSS, CBF-ASPECTS, and CBV-ASPECTS demonstrated good areas under the curve for predicting various outcomes in AIS patients: 0.833, 0.874, and 0.899, respectively. The optimal thresholds for CBF-ASPECTS and CBV-ASPECTS are 8 and 6, respectively, both exhibiting a sensitivity of 0.818. In contrast, the optimal threshold for baseline NIHSS is 3, demonstrating a relatively low sensitivity of 0.727. The specificity values ranged from 0.795 for CBF-ASPECTS to 0.886 for CBV-ASPECTS, with the baseline NIHSS falling in between at 0.864. The results presented above demonstrate that both the baseline NIHSS and CBF-ASPECTS or CBV-ASPECTS were effective in identifying potential poor outcomes in AIS patients, with CBV-ASPECTS displaying relatively better predictive capability.

By integrating baseline NIHSS with perfusion indicators CBF-ASPECTS and CBV-ASPECTS, the combined area under the curve improved to 0.963, enhancing both sensitivity and specificity to 0.909. The AUC of this combined indicator demonstrates a statistically significant improvement over the baseline NIHSS. It is evident that enhancing the perfusion ASPECTS information from multi-PLD ASL improves the predictive accuracy for patient prognosis in clinical practice. Simultaneously, we observed no statistical variance between the AUC of the combined indicator and the AUC of CBV-ASPECTS. The results suggested that CBV-ASPECTS can provide a similar predictive efficacy as the baseline NIHSS combined with CBF/CBV-ASPECTS. In essence, this suggested that in scenarios with insufficient clinical data, evaluating ASPECTS based on multi-PLD ASL could still provide valuable insights into the prognosis of stroke patients. The discovery is of great significance. As it is common in clinical practice to not acquire accurate baseline clinical neurological function assessment data for patients.

There are several shortcomings in this study. Firstly, the sample size of the trial was relatively small. Secondly, this study only included AIS patients with non-major vessel embolization who were treated with conservative medical therapy. Future studies should include larger cohorts and patients treated with thrombolysis or thrombectomy to better generalize the findings. Thirdly, the factors affecting the long-term prognosis of stroke are complex and diverse, and this study standardized the baseline clinical characteristics as much as possible by including only AIS patients with first-ever unilateral anterior circulation infarction and standard medical clinical treatment. Fourthly, the clinical prognostic value of multi-PLD ASL has not been directly compared with other advanced imaging techniques. In future research, we will conduct a direct comparison of the prognostic value of multi-PLD ASL with dynamic susceptibility contrast MRI, QSM or positron emission tomography imaging in AIS patients to further establish the utility of multi-PLD ASL.

## 5 Conclusion

In conclusion, the ASPECTS based on multi-PLD ASL is a valuable tool for identifying independent prognostic indicators and assessing clinical outcomes in AIS patients. CBF-ASPECTS and CBV-ASPECTS, as independent prognostic indicators, are significantly associated with clinical outcomes. Furthermore, the baseline NIHSS combined with CBF-ASPECTS and CBV-ASPECTS enhances the predictive efficacy of clinical outcomes in AIS patients. CBV-ASPECTS alone can offer a comparable predictive efficacy to the combination. Thus, the incorporation of multi-PLD ASL into routine clinical evaluation may significantly enhance the precision of prognosis, thereby allowing for more tailored interventions in AIS patients.

## Data Availability

The datasets presented in this article are not readily available because, the raw data supporting the conclusions of this article will be made available by the authors, without undue reservation. Requests to access the datasets should be directed to Qingqing Li, lsh946901143@163.com.
